# The network of cardiac K_IR_2.1: its function, cellular regulation, electrical signaling, diseases and new drug avenues

**DOI:** 10.1007/s00210-024-03116-5

**Published:** 2024-04-29

**Authors:** Encan Li, Marcel A. G. van der Heyden

**Affiliations:** Department of Medical Physiology, Division Heart & Lungs, University Medical Center Utrecht, Yalelaan 50, 3584 CM Utrecht, Netherlands

**Keywords:** K_IR_2.1 channels, I_K1_, Electrical signaling, Cell trafficking, Cell communication, Cardiovascular diseases, Future developments, Pharmacology

## Abstract

The functioning of the human heart relies on complex electrical and communication systems that coordinate cardiac contractions and sustain rhythmicity. One of the key players contributing to this intricate system is the K_IR_2.1 potassium ion channel, which is encoded by the *KCNJ2* gene. K_IR_2.1 channels exhibit abundant expression in both ventricular myocytes and Purkinje fibers, exerting an important role in maintaining the balance of intracellular potassium ion levels within the heart. And by stabilizing the resting membrane potential and contributing to action potential repolarization, these channels have an important role in cardiac excitability also. Either gain- or loss-of-function mutations, but also acquired impairments of their function, are implicated in the pathogenesis of diverse types of cardiac arrhythmias. In this review, we aim to elucidate the system functions of K_IR_2.1 channels related to cellular electrical signaling, communication, and their contributions to cardiovascular disease. Based on this knowledge, we will discuss existing and new pharmacological avenues to modulate their function.

## Introduction

The term “inward rectification" was first introduced in 1949 to describe a phenomenon where ion channels preferentially allow potassium current to flow into the cell rather than out of it even with an opposite driving force (Katz 1949; Nichols and Lopatin 1997). This current was described as an “inward rectifier potassium current (I_K1_)”. The channels which produce the current are currently known as inward-rectifier potassium (K_IR_) channels (Li and Dong 2010; Lopatin and Nichols 2001). Since their discovery, detailed knowledge on these channels accumulated. Expression patterns were established, rectification mechanisms deciphered, and the molecular structure is now resolved. Functional I_K1_ channels (K_IR_2.1, K_IR_2.2, and K_IR_2.3) were found by patch clamp in nearly all the cardiac myocytes (Anumonwo and Lopatin 2010). K_IR_2.1 is predominantly expressed in Purkinje fibers and human ventricular cardiomyocytes, K_IR_2.2 shows a lower degree of expression when compared with K_IR_2.1 in ventricles, and K_IR_2.3 exists relatively more in the human atria (Anumonwo and Lopatin 2010). K_IR_2.2 or K_IR_2.3 subunits can form heterotetramers with K_IR_2.1 to modulate I_K1_ (Cui et al. 2021; Panama et al. 2007, 2010; Zobel et al. 2003). Cardiac I_K1_ is mainly composed of K_IR_2.1 and K_IR_2.2 heterotetramers (Zobel et al. 2003). K_IR_2.2 subunits contributed more strongly to the single-channel conductance but with a significantly shorter opening time when compared with K_IR_2.1 (Panama et al. 2010). Channels that contain 2 or more K_IR_2.2 subunits showed similar conductance with homomeric K_IR_2.2 channels (Panama et al. 2010). When one K_IR_2.3 subunit was added to a K_IR_2.1 channel, the activation kinetics slowed by approximately threefold, with greater slowing when more K_IR_2.3 subunits were subsequently added (Panama et al. 2007). The inward rectification behavior mainly results from pore blocking by intracellular substances, such as magnesium ions (Mg^2+^) and polyamines (Baronas and Kurata 2014; Ishihara et al. 2009). I_K1_ acts in concert with many other ion channels and some forms of co-regulation at the cell biological level is present.

K_IR_2.1 channels play important roles in maintaining cells' resting membrane potential (RMP), regulating cell excitability, and participating in various physiological processes (Cui et al. 2021; Dhamoon and Jalife 2005; Li and Yang 2023; Reilly and Eckhardt 2021). Dysfunctional K_IR_2.1 channels will disrupt the hearts normal electrical activity, leading to irregular heart rhythms and potentially life-threatening arrhythmias (Crotti et al. 2020; Reilly and Eckhardt 2021; Van Der Schoor et al. 2020; Zangerl-Plessl et al. 2019). This review focuses on the importance of K_IR_2.1 channels in mediating cellular electrical signaling, cell communication, and their involvement in cardiovascular diseases. Now, the gained knowledge impacts our understanding of K_IR_2.1 pharmacology and provides new insights for their drug-design.

## K_IR_2.1 cellular electrical signaling

### Structure of the K_IR_2.1 channel

**Fig. 1:**
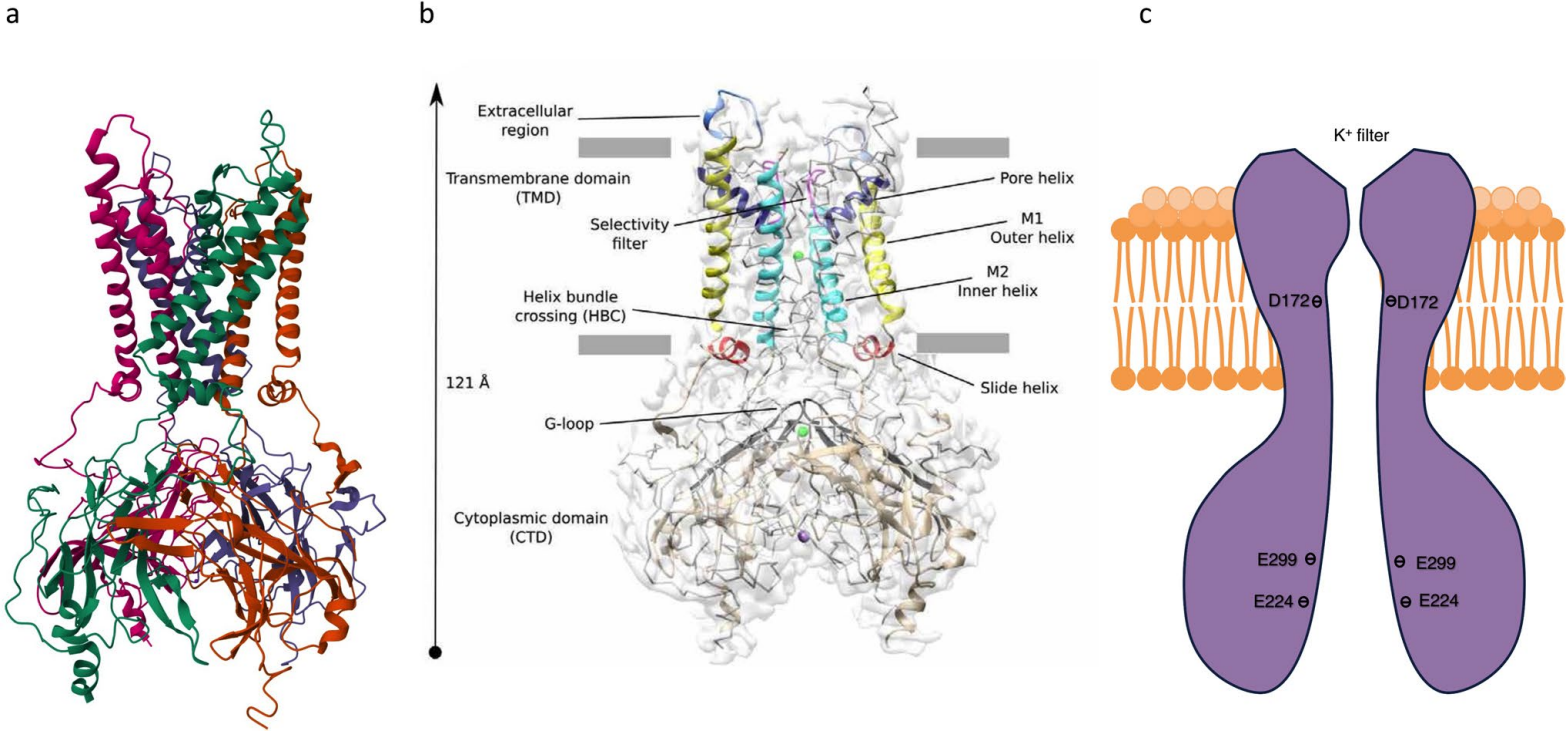
Structure of the K_IR_2.1 channel. **a** and **b** Side view of the human K_IR_2.1 atomic structure fitted in the cryo-EM map (Fernandes et al. 2022a, b) (reproduced with approval of the original authors). (**c**) Schematic representation of the K_IR_2.1. D172, E299, and E224 are polyamine binding sites which are negatively charged. D172 has a strong binding affinity with polyamines, while E299 and E224 show a weak binding affinity with polyamines

The first complete K_IR_2.1 channel sequence, encoded by the *KCNJ2* gene, from the mouse, was cloned in 1993 (Kubo et al. 1993). Nearly thirty years later, the first cryo-electron microscopy derived structure of the human K_IR_2.1 channel was presented (Fig. 1a, b) (Fernandes et al. 2022a, b). K_IR_2.1 channels are formed by an interaction of four K_IR_2.1 proteins and contain both a transmembrane domain (TMD) and a cytoplasmic domain (CTD) (Fernandes et al. 2022b). The TMD of each protein is composed of 2 transmembrane helices M_1_ and M_2_ separated by a selectivity filter, containing the K^+^-channel signature sequence (T-X-G-Y/F-G). Additionally, there are two short helical components known as the slide helix and the pore helix, along with the M_1_ and M_2_ (Fig. 1b) (Fernandes et al. 2022b). The CTD is composed of the amino (NH_2_)- and carboxy (COOH)-terminal regions located on the cytoplasmic side forming a long inner vestibule that serves as an extension of the channel pore (Fernandes et al. 2022b; Lu et al. 1999). Around the membrane face of the CTD, an intrinsically flexible loop named “G-loop” exists which forms the narrowest portion of the ion conduction pathway (Fig. 1b) (Fernandes et al. 2022b; Hibino et al. 2010). Along the TMD and CTD, there are many channel activator and inhibitor binding sites that interfere with the open and closed state of the channel and serve the function of K_IR_2.1 as an inward rectification potassium channel (Hibino et al. 2010). Some of these sites might serve as targets in drug development.

### Physiological functions of K_IR_2.1 channels and the cardiac action potential

**Fig. 2:**
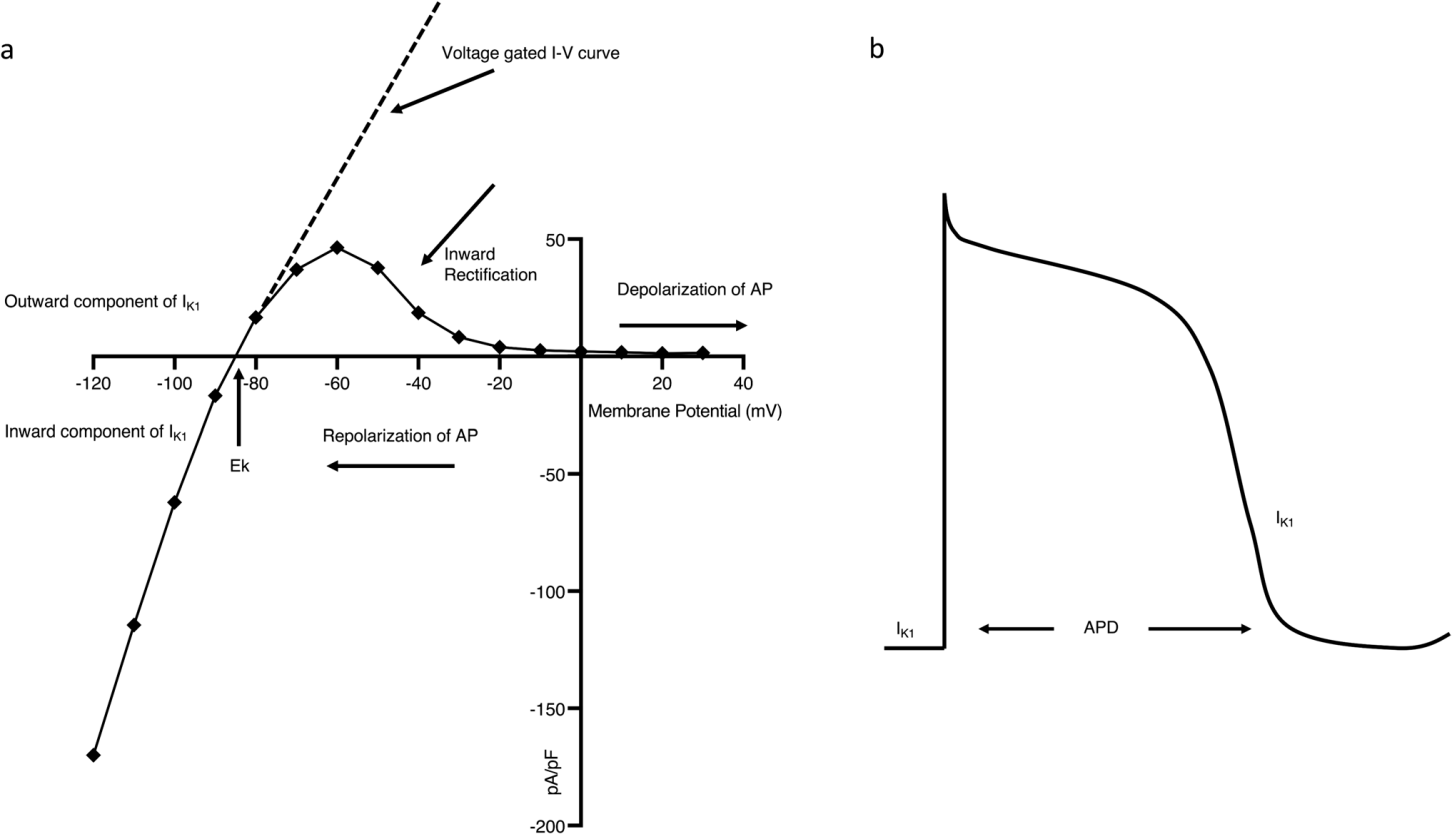
A typical example of I_K1_ recording in a KWGF cell and its effect on AP. KWGF cells are Human embryonic kidney (HEK)-293 cells that stably express C-terminal GFP (Green fluorescent protein)-tagged murine K_IR_2.1 (De Boer et al. 2006; Li et al. 2023). **a** I_K1_ current/voltage (I/V) relationship showing a reversal potential at approximately -85 mV and strong rectification at voltages between -60 and + 30 mV. The current above the X-axis is the outward component, and the current below the X-axis is the inward component. The dotted line represents the I-V curve without the rectification. **b** Contribution of I_K1_ to the cardiac AP. Temporal changes in I_K1_ could determine the duration of AP (APD), as it blocks during the depolarization phase and increases in the final repolarization phase

K_IR_2.1 channels play a vital role in mediating cellular electrical signaling in various tissues, including the cardiovascular system (De Boer et al. 2010a). In resting cardiomyocytes, the potassium equilibrium potential, Ek, is slightly more negative than the resting membrane potential. Therefore, at the level of resting membrane potential, K_IR_2.1 channels exhibit a certain outward current. This outward current can clamp the membrane potential at a more negative level, which helps to stabilize the resting membrane potential and regulate excitability. This outward current tends to zero after further depolarization because of the inward rectification (Hibino et al. 2010). The lack of outward conductance of I_K1_ at positive potentials permits maintenance of a positive membrane potential established by several depolarizing currents (Hibino et al. 2010; Kubo et al. 1993; Lopatin and Nichols 2001). Then time dependent closure of depolarizing channels and concomitant opening of voltage-gated potassium channels allows a rapid efflux of K^+^ ions, leading to a repolarization (Grider et al. 2023). K_IR_2.1 channels play a role in the terminal phase of the cardiac action potential (AP) (Reilly and Eckhardt 2021). When the membrane potential starts to become more negative again, the K_IR_2.1 channels gradually recover their conductance (Hibino et al. 2010). This recovery allows a relatively large outward flow of potassium ions to pass through K_IR_2.1 channels which contributes to shortening of the cells’ action potential duration (APD) (Hibino et al. 2010). During these phases in the repolarization process, the I_K1_ inward current component will prevent a repolarization overshoot. Hence, the I_K1_ current contributes to the normal duration of the AP and stability of the resting membrane potential (Fig. 2). The I_K1_ activity helps to maintain the heart's normal electrical activity and reduces the likelihood of arrhythmias. Loss- or gain-of-function mutations or acquired dysregulation of the channel lead to various pathological conditions, these mutations may impair the channel's ability to properly open or close, resulting in modified ion conductance and disrupted cellular excitability.

### Opening and closing mechanisms of K_IR_2.1 channels

K_IR_2.1 channels exhibit unique opening and closing mechanisms that regulate their activity and play a crucial role in cellular physiology. The channel opening and closing mechanisms of K_IR_2.1 channels involve various factors, including membrane voltage, intracellular polyamines, lipids, modulatory proteins, several cations, and others (Hibino et al. 2010).

At membrane potentials negative to the reversal potential (Ek), the channels exhibit a high open probability, facilitating the influx of potassium ions (Anumonwo and Lopatin 2010; Dhamoon and Jalife 2005; Li and Dong 2010). Conversely, the channels undergo pore block at membrane potentials positive to the voltage threshold, resulting in closure and diminished ion conductance (Anumonwo and Lopatin 2010; Dhamoon and Jalife 2005; Li and Dong 2010). The driving force behind ion movement is determined by a combination of an electrical and potassium gradient over the plasma membrane (Gadsby 2009). Changes in membrane potential due to depolarization or hyperpolarization led to alterations in the electric driving force (membrane potential Vm—reversal potential Ek), the altered electric driving force works on the chemical driving force, subsequently influencing K^+^ ion movement in or out of the cell. Therefore, it can be explained that even though K_IR_2.1 channels are not voltage-gated by itself, they can still be influenced by the membrane potential.

K_IR_2.1 channels exhibit a constant permeability when the inward or slightly outward potassium driving force is present. However, when the driving force becomes significantly outward, K^+^ permeability declines rapidly. The mechanism underlying this driving force-induced permeability change primarily involves the blockage of outward potassium current by intracellular Mg^2+^ or polyamines such as spermine and spermidine (Ficker et al. 1994; Ishihara et al. 2009; Nichols and Lee 2018). These positively charged substances bind to negatively charged amino acids within the pore of the K_IR_2.1 channel, thereby reducing the conductance of outward currents (Fujiwara and Kubo 2006). Early studies showed that Mg^2+^ dependent I_K1_ block is the cause of inward rectification, but increasing amount of studies now proved that spermine is the main factor responsible for inward rectification, followed by spermidine, putrescine and then Mg^2+^ (Anumonwo and Lopatin 2010; Ficker et al. 1994; Kubo 1996; Lopatin et al. 1994; Nichols and Lee 2018). This K_IR_2.1 channel block occurs in a two-step process. The initial step, which is weakly voltage-dependent, involves the entry and interaction of polyamines into the K_IR_2.1 channel pore (Anumonwo and Lopatin 2010; Nichols and Lee 2018). This interaction occurs at a specific site of negatively charged amino acids (E224 and E299) located in the C-terminus of the channel (Fig. 1c) (Anumonwo and Lopatin 2010; Kubo and Murata 2001; Nichols and Lee 2018). The subsequent step, which is more strongly voltage-dependent, involves the movement of polyamines to a deeper binding site, in the TMD, at the D172 residue (Fig. 1c) (Anumonwo and Lopatin 2010; Kubo and Murata 2001). During membrane hyperpolarization, the time-dependent activation of strong inward rectifiers reflects the exit of polyamines from the pore (Ishihara et al. 1996; Ishihara and Ehara 2004).

K_IR_2.1 channels engage in interactions with diverse regulatory proteins, including protein kinase A (PKA), protein kinase C (PKC), and Phosphatidylinositol-4,5-bisphosphate (PIP_2_) (D'avanzo et al. 2010; Karschin 1999; Reilly and Eckhardt 2021; Trum et al. 2020; Xie et al. 2008). For example, an earlier study showed that the open probability of a recombinant K_IR_2.1 and K_IR_2.3 is inhibited by both PKA and PKC mediated phosphorylation (Karschin 1999). PIP_2_, a crucial lipid constituent of the plasma membrane, acts as a positive modulator of K_IR_2.1 channels by binding to specific sites within the channel structure, thereby regulating the channel and enhancing its open probability (D'avanzo et al. 2010; Fernandes et al. 2022b; Li et al. 2015; Ruddiman et al. 2023; Xie et al. 2008).

### Regulation of I_K1_ by different cations

Three physiological relevant cations, i.e. K^+^, Na^+^, Ca^2+^, and the poisonous Ba^2+^ are well known to affect the K_IR_2.1 channel function (Anumonwo and Lopatin 2010; Bhoelan et al. 2014; Hibino et al. 2010). Elevating the concentration of extracellular K^+^ ([*K*^+^]_o_) tends to enhance I_K1_ (Chang et al. 2010; Ishihara 2018; Kubo 1996; Liu et al. 2011). For the inward component, this is easy to explain since a higher [*K*^+^]_o_ increases the chemical driving force for K^+^ flow into the cells. But with regard to the outward component, several hypotheses have been raised. Some researchers think that the elevated [*K*^+^]_o_ activates the channel, allowing more K^+^ flow out of cells, thus interfering with the channel’s open probability (Pennefather et al. 1992). Some prefer the view that elevated [*K*^+^]_o_ weakens the polyamine or Mg^2+^-induced rectification of the channel so that the peak I_K1_ current is increased (Kubo 1996). Some overturned these two hypotheses, and they proved that the open probabilities of the channel and spermine-binding kinetics were not interfered when the [*K*^+^]_o_ increases at a constant driving force, but the conductance of the channel was increased (Liu et al. 2011). In addition, some researchers proved that the [*K*^+^]_o_ increase in I_K1_ is not caused by activating the channel but is caused by physiologically relevant competition from impermeant extracellular Na^+^ or Ga^2+^ (Chang et al. 2010; Ishihara 2018). Increased concentrations of extracellular Na^+^ ([Na^+^]_o_) reduce the outward I_K1_ due to pore blocking and surface charge effect (Chang et al. 2010; Ishihara 2018).

Many studies agree that I_K1_ is Ca^2+^-sensitive, but the modulatory effects of Ca^2+^ on I_K1_ are controversial (Nagy et al. 2011, 2013; Wagner et al. 2009). In some cases, I_K1_ was shown to decrease with elevated intracellular or extracellular Ca^2+^ ([Ca^2+^]_i/o_) (Chang et al. 2010; Fauconnier et al. 2005; Matsuda and Cruz Jdos 1993; Mazzanti and Defelice 1990; Zaza et al. 1998). In ventricular myocytes isolated from failing rat hearts, I_K1_ was observed to decrease because of the blocking effect by elevated [Ca^2+^]_i_ (Fauconnier et al. 2005). Similar to [Na^+^]_o_, the increased [Ca^2+^]_o_ was reported to reduce I_K1_ by blocking the channel pore and the effect of altered surface charge (Chang et al. 2010). Calcium/Calmodulin-dependent protein kinase II (CaMKII), an enzyme that relies on elevated [Ca^2+^]_i_ for its activation, regulates I_K1_ (Ma et al. 2021; Nagy et al. 2013; Wagner et al. 2009). Chronic activation of CaMKII was reported to downregulate K_IR_2.1 mRNA expression and decrease I_K1_ in a mouse model (Wagner et al. 2009). In addition, I_K1_ also shows an increase with rising [Ca^2+^]_i_ in some other cases (Nagy et al. 2011, 2013). For example, Ca^2+^ influx during exercise was shown to increase I_K1_ (Nagy et al. 2011). Another study showed that elevated [Ca^2+^]_i_ results in an increase in I_K1_ in isolated dog ventricular cardiomyocytes, and I_K1_ was significantly reduced by inhibiting CaMKII (Nagy et al. 2013). This [Ca^2+^]_i_-dependent augmentation of I_K1_ shortened the repolarization phase of the AP, suggesting that it could serve as a protective mechanism against cardiac arrhythmias induced by the calcium overload (Nagy et al. 2013). Therefore, the influence of Ca^2+^ on I_K1_ appears to be dependent on the specific experimental conditions and cellular context, leading to conflicting findings among studies.

Ba^2+^ is an effective K_IR_2.1 channel blocker leading to a decrease in the I_K1_ current, and this block is both voltage and time-dependent (Imoto et al. 1987). Blockage is achieved by a Ba^2+^ ion entering and blocking the pore at the selectivity filter. At more negative membrane potentials, Ba^2+^ has a strong sensitivity to the channel and causes stronger and more rapid block than when the membrane potential becomes more positive (Imoto et al. 1987). Cs^+^ and Sr^2+^ also exhibit full inhibitory activity immediately at the beginning of each application period, but these 2 blockers were shown less potent than Ba^2+^ (Sanson et al. 2019). A recent study revealed an additional mechanism of blocking apart from pore obstruction of external Ba^2+^ and Cs^+^ (Gilles 2022). The work suggests interaction of the ions to an extracellular side of the channel. Furthermore, blockage is independent of the K^+^ ion flux (Gilles 2022).

## K_IR_2.1 channels in their cellular context

### Channel trafficking

**Fig. 3:**
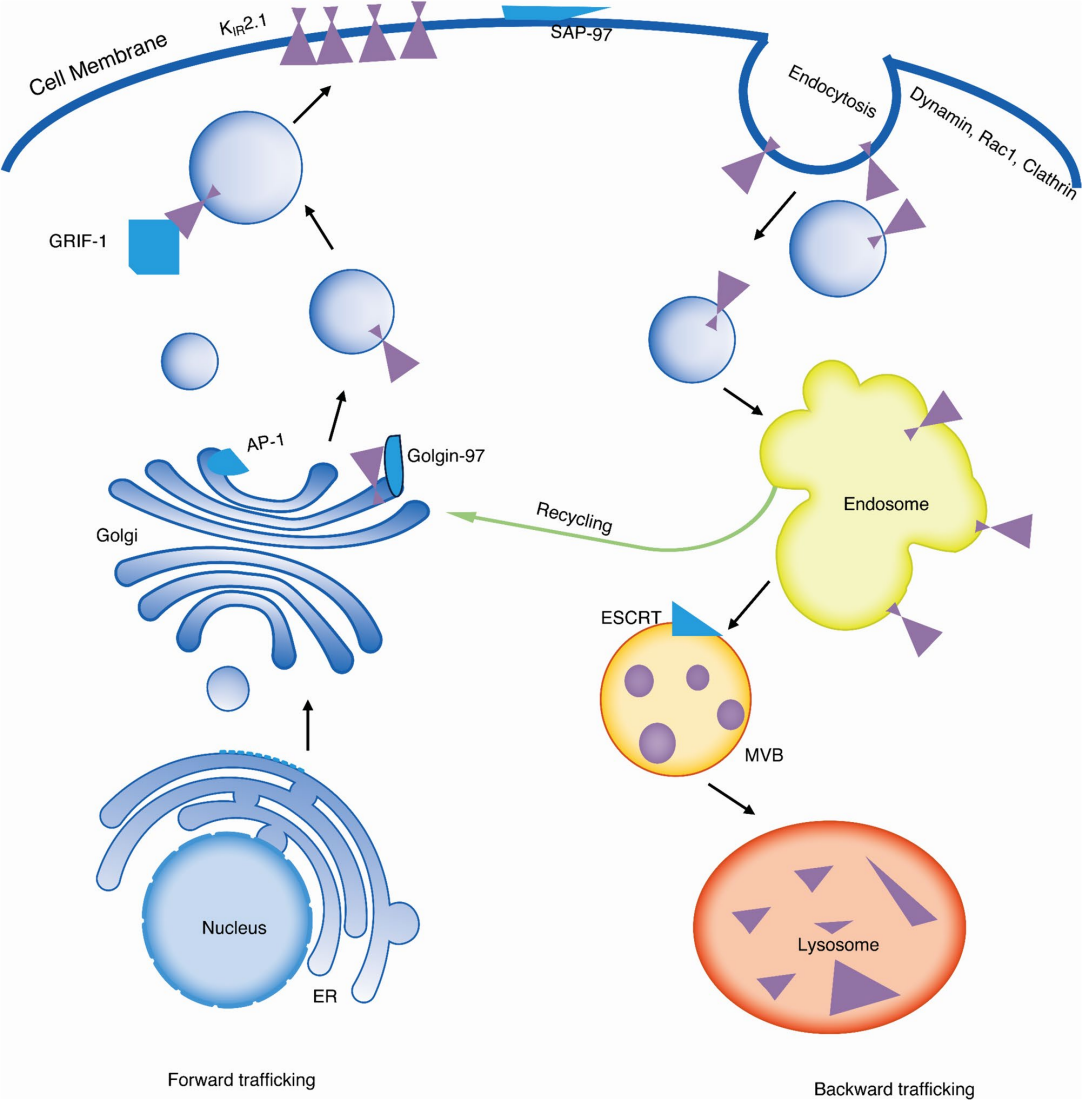
Schematic representation of intracellular trafficking pathways of K_IR_2.1 channels. Arrows indicate the trafficking routes for K_IR_2.1. ER, endoplasmic reticulum; MVB, multivesicular bodies; AP-1, Golgin-97, GRIF-1, SAP-97, Rac-1, and ESCRT are regulators during the process of K_IR_2.1 proteins trafficking

Channel trafficking refers to the process by which channel proteins are transported to their appropriate cellular destinations within a cell or between different cellular compartments (Steele et al. 2007). In general, ion channel trafficking can be divided into forward trafficking (towards the plasma membrane), backward trafficking (removed from the plasma membrane), and recycling that couples backward to forward trafficking mechanisms (De Git et al. 2013; Hager et al. 2021) (Fig. 3). It is an essential cellular mechanism that ensures proteins are delivered to the correct location for their proper function. Proteins are synthesized in the endoplasmic reticulum (ER), and only properly folded and assembled channels are exported from the ER to the Golgi apparatus (Steele et al. 2007). The field of cystic fibrosis, in which mutations in the affected chloride channels result often in aberrant channel expression at the plasma membrane, demonstrated the clinical efficacy and specificity of pharmacological chaperones to restore normal channel function (Gramegna et al. 2021).

The K_IR_2.1 protein has an ER export sequence in the C-terminus (^374^FCYENEV^380^, numbers based on human sequence) and a Golgi export sequence in the N-terminus (^44^RSRFVK^49^) which play a role along the intracellular forward trafficking route (Hofherr et al. 2005; Ma et al. 2001, 2011; Stockklausner et al. 2001; Stockklausner and Klocker 2003). Disruption of the ^374^FCYENEV^380^ motif (GFPK_IR_2.1-E377/379A) resulted in an accumulation of K_IR_2.1 channel proteins in the ER and led to a decrease in its presence in both the Golgi and the plasma membrane (Ma et al. 2001; Stockklausner and Klocker 2003). The S369X mutant led to a premature stop codon at S369, causing a loss of 59 amino acids in the C-terminal (Doi et al. 2011). Individuals carrying the S369X mutation will lose the ER export motif and be diagnosed with Andersen-Tawil syndrome (ATS) (Doi et al. 2011). Disruption of the ^44^RSRFVK^49^ motif leads to an accumulation of K_IR_2.1 channel proteins within the Golgi and causes a significant reduction of properly folded K_IR_2.1 anchoring on the cell membrane (Hofherr et al. 2005; Ma et al. 2011; Stockklausner and Klocker 2003). This Golgi export sequence can form a recognition site for the clathrin adaptor proteins (AP-1), thereby marking K_IR_2.1 channel proteins for packaging into different clathrin-coated vesicle (Hofherr et al. 2005; Ma et al. 2011). Besides the N-terminal sequence, a C-terminal ATS mutation (K_IR_2.1△314–315) blocks Golgi exit similarly (Ma et al. 2011). The colocalization of this mutant channel with *trans*- and *cis*-Golgi markers suggests that the mutation blocks channel protein trafficking out of the Golgi (Ma et al. 2011).

Disruptions in protein trafficking can have severe consequences for cellular function and can lead to various diseases. Loss-of-function mutation associated and acquired disruptions in normal K_IR_2.1 protein trafficking can result in altered membrane expression of K_IR_2.1 channels resulting in, for example, abnormal repolarization (De Git et al. 2013; Hager et al. 2021). This can contribute to the development of ATS and an increased risk of arrhythmias (Hibino et al. 2010). Proper K_IR_2.1 channel trafficking is also important in other systems besides the cardiovascular system (Akyuz et al. 2022; Binda et al. 2018). For example, decreased expression of K_IR_2.1 channels in neurons can result in altered membrane potentials and increased neuronal excitability, these changes can contribute to the development of epilepsy (Akyuz et al. 2022). In contrast, enhanced expression of K_IR_2.1 channels was involved in the autism spectrum disorder (ASD) (Binda et al. 2018).

K_IR_2.1 channel trafficking can be regulated by various signals and factors. Interactions with other proteins can lead to altered trafficking or degradation of the channel. The hypomorphic K_IR_2.1Δ314–315 mutation of the K_IR_2.1 channel disrupts Golgi trafficking by deficient AP-1 binding, leading to the development of ATS (Ma et al. 2011). In the Golgi apparatus, Golgin-97 which belongs to membrane and cytoskeleton tethers helps capture the K_IR_2.1-containing vesicles to Golgi and facilitates K_IR_2.1 transport into AP-1 associated vesicles (Hager et al. 2021; Taneja et al. 2018). Even though Golgin-97 is necessary for the forward trafficking of the K_IR_2.1 channel proteins, it also mediates the retrograde transport of endosomes to the Golgi (Shin et al. 2017). The γ-aminobutyric acid type A receptor interacting factor-1 (GRIF-1) plays a role in the forward trafficking by binding with the C-terminus of K_IR_2.1, then promoting the trafficking of vesicles and facilitating the anchoring of K_IR_2.1 channel protein on the cell membrane (Grishin et al. 2006; Hager et al. 2021). SAP-97 which is a membrane-associated scaffolding protein, regulates the Na_V_1.5/K_IR_2.1 complex, leading to a decrease in the internalization of K_IR_2.1 (Milstein et al. 2012). Ras-related C3 botulinum toxin substrate 1 (Rac1) showed strong specificity with K_IR_2.1 channels, as K_IR_2.2 and K_IR_2.3 channels were not involved in the regulation by Rac1 (Boyer et al. 2009). Data suggested that inhibiting Rac1 resulted in approximately a twofold increase in K_IR_2.1 channel expression by interfering with endocytosis, likely via a dynamin-dependent pathway (Boyer et al. 2009). The endosomal sorting complex required for transport (ESCRT) was required for the lysosome-dependent degradation of K_IR_2.1 in human cells and regulates the level of K_IR_2.1 at the cell membrane (Hager et al. 2021; Kolb et al. 2014). Clathrin-mediated endocytosis and late endosomal or lysosomal activities are also critical for the degradation of K_IR_2.1 channels (Jansen et al. 2008; Li et al. 2023; Varkevisser et al. 2013). Our previous study proved that propafenone causes intracellular accumulations of K_IR_2.1 most likely by inhibiting the function of the late endosome (Li et al. 2023). Intracellular signaling pathways impact the trafficking of K_IR_2.1 channels, for example, the Ras-MAPK pathway modulates the I_K1_ current by altering the channel density on the cell membrane (Giovannardi et al. 2002). In addition, functional actin and tubulin cytoskeleton systems are crucial for the forward trafficking of K_IR_2.1 channels, and in turn functional membrane expression and anchoring of the K_IR_2.1 channel also regulate the actin filament dynamics (Li et al. 2022; Wu et al. 2020). The initial backward trafficking depends on a functional dynamin system (Li et al. 2022).

### Coregulation of functional pairs

From a physiological viewpoint different ion channels have to coordinate their activity, for example to generate proper action potential characteristics. To this end, several ion channels are found to make functional pairs, also referred to as channelosomes (Gutierrez et al. 2024). Sodium inward current (I_Na_) and I_K1_ current are two important ionic currents that control the ventricular excitability (Varghese 2016). Strong evidence showed the existence of reciprocal modulations between Na_V_1.5 and K_IR_2.1 channels (Dago et al. 2022; Goversen et al. 2016; Li et al. 2021b; Macías et al. 2022; Matamoros et al. 2016; Milstein et al. 2012; Perez-Hernandez et al. 2018; Ponce-Balbuena et al. 2018; Utrilla et al. 2017; Varghese 2016). K_IR_2.1 and Na_V_1.5 channels physically interact and form the macromolecular complexes (K_IR_2.1- Na_V_1.5 channelosomes) during transportation from sarcoplasmic reticulum (SR) to Golgi, and then trafficking together to the cell membrane (Gutierrez et al. 2024; Ponce-Balbuena et al. 2018). Na_V_1.5 channel proteins can reduce the internalization of K_IR_2.1 channel proteins to promote the channel presence at the cell membrane (Milstein et al. 2012). Trafficking defects in the Na_V_1.5 channel will cause a decreasing I_K1_ in addition to I_Na_ (Perez-Hernandez et al. 2018; Reilly and Eckhardt 2021). Similarly, trafficking defects of K_IR_2.1 lead to a down-regulation of Na_V_1.5 expression and current density (Macías et al. 2022). Increased expression of Na_V_1.5 channels concurrently induces the upregulation of the K_IR_2.1 channel expression (Milstein et al. 2012). In a human in vitro cardiomyocyte myocardial infarction model, a decreased Na_V_1.5 and K_IR_2.1 protein expression accompanied by reductions in I_Na_ and I_K1_ was observed, and functional expression of both channels could be restored by liver-derived fibroblast growth factor 21 (Li et al. 2021b). The reciprocal modulation between K_IR_2.1 and Na_V_1.5 relies on a specific C-terminal PDZ-binding domain located in K_IR_2.1 and a PDZ-like binding domain located at the N-terminus of the Na_V_1.5 channel (Matamoros et al. 2016). Dysfunction of Na_V_1.5 and K_IR_2.1 channelosomes is associated with severe cardiac diseases, such as ATS, Short QT syndrome type 3 (SQT3), Brugada syndrome and Duchenne muscular dystrophy (Gutierrez et al. 2024). However, the effect of K_IR_2.1 channel expression on Na_V_1.5 channels remains controversial and might be stimulus specific. For example, our previous study demonstrated that propafenone enhances the expression level of K_IR_2.1 channel proteins without interfering with Na_V_1.5 channel expression (Li et al. 2023; Milstein et al. 2012). Another study that is inconsistent with the reciprocity modulation demonstrates that in synapse-associated protein-97 (SAP-97) knockout mouse cardiomyocytes, I_K1_ was decreased but I_Na_ was not altered (Gillet et al. 2015).

Gap junctions are specialized structures that allow direct communication and electrical coupling between cells, which is critical for synchronized activity in tissues like the heart (Gao et al. 2015). Gap junctions facilitate the conduction of APs from one myocyte to another (Veeraraghavan et al. 2014). Functional gap junctions exist of connexin proteins (Zhang et al. 2018). The major gap junction connexin between ventricular cardiomyocytes is Connexin-43 (Cx-43) (Gao et al. 2015). Some studies demonstrated that the expression levels of Cx-43 and K_IR_2.1 channel proteins altered synchronously after treatment (Lee et al. 2015; Qian et al. 2021; Raad et al. 2021; Zhang et al. 2018, 2013b). For example, the expression levels decreased in model rat myocardial tissues and were elevated after pretreatment with pinocembrin (Zhang et al. 2018). The relationship between Cx-43 and K_IR_2.1 channels probably lies in their contribution to the overall electrical behavior of cardiac tissues. A study transfected the gene of K_IR_2.1 channel (*KCNJ2*), Na_V_1.5 channel (*SCN5A*), and the Cx-43(*GJA1*) in HEK-293 T cells proved that overexpression of Cx-43 in these transfected cells shows enhanced intercellular coupling and permits rapid AP propagation (Kirkton and Bursac 2011). Another study transfected varying ratios of K_IR_2.1 and bacterial sodium channel (Na_V_D) with and without Cx-43 in cells, found more Ca^2+^ responses were generated in cells expressed with Cx-43 (Thomas and Hughes 2020). Cx-43 is a Ca^2+^-dependent channel, and I_K1_ is also Ca^2+^-sensitive (Lurtz and Louis 2007; Nagy et al. 2011). In this perspective, perhaps Ca^2+^ is the bridge between K_IR_2.1 and Cx-43. However, in some cases of human AF, the K_IR_2.1 channel expression and I_K1_ current were increased without interfering with the localization and expression of the Cx-43 (Girmatsion et al. 2009). These different alterations may suggest an overlapping regulatory pathway between the channel of K_IR_2.1 and Cx-43.

## K_IR_2.1 related cardiovascular diseases and pharmacological avenues

**Fig. 4:**
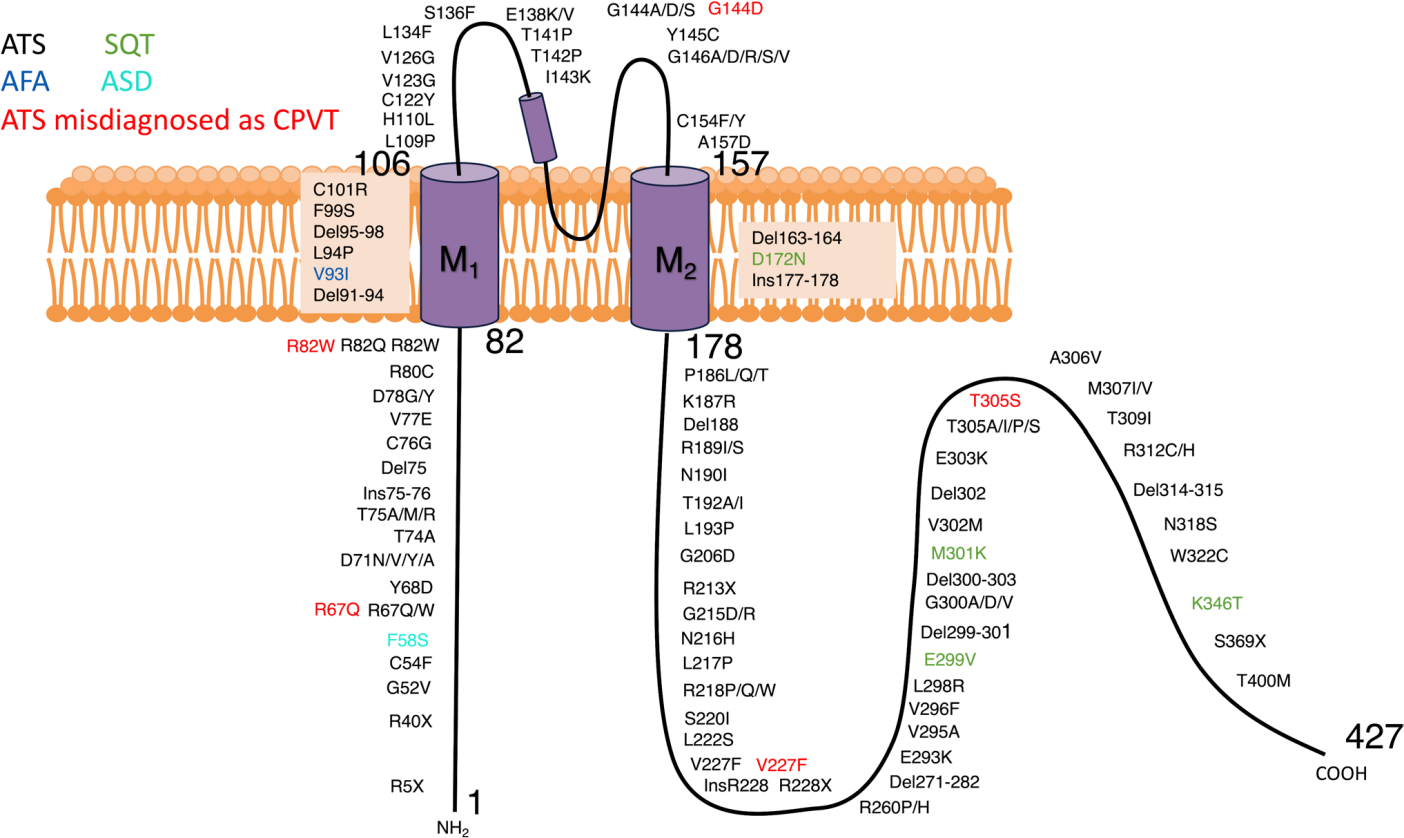
Representations of disease-associated mutations on K_IR_2.1 protein. Loss-of-function mutation associated with ATS (Anumonwo and Lopatin 2010; Beverley and Pattnaik 2022; Fernlund et al. 2013; Fukumura et al. 2019; Kimura et al. 2012; Moreno-Manuel et al. 2023; Nguyen et al. 2013; Obeyesekere et al. 2011; Šinkovec et al. 2013; Van Ert et al. 2017; Villar-Quiles et al. 2022; Vivekanandam et al. 2022; Yim et al. 2021) shown in black, and gain-of-function mutations associated with SQT (Ambrosini et al. 2014; Deo et al. 2013; Hattori et al. 2012; Priori et al. 2005), FAF (Xia et al. 2005), and ASD (Binda et al. 2018) are depicted in green, blue, and brown font respectively. Loss of function mutations which were misdiagnosed as CPVT (Walsh et al. 2022) are in red. “Ins” is for insertion and “Del” is for deletion. ATS, Anderson-Tawil syndrome; SQT, Short QT Syndrome; FAF, Familial Atrial Fibrillation; ASD, autism spectrum disorder; CPVT, Catecholaminergic Polymorphic Ventricular Tachycardia

K_IR_2.1 channel activity directly affects the cardiac electrical stability of the human heart (Hibino et al. 2010; Li and Dong 2010; Reilly and Eckhardt 2021). The gain-of-function and loss-of-function of the K_IR_2.1 channel can both cause different kinds of arrhythmias (Hibino et al. 2010). Some of the mutations on the *KCNJ2* gene associated with cardiovascular diseases are shown in Fig. 4. Besides genetic causes, impaired K_IR_2.1 channel function resulting from drugs, electrolyte abnormalities, etc. can also lead to the development of some acquired diseases (Kim 2014; Maruyama et al. 2011). The following K_IR_2.1 channel diseases are associated with the heart.

### The Anderson-Tawil syndrome

The Anderson-Tawil syndrome (ATS), characterized by periodic paralysis, cardiac arrhythmias, and dysmorphic features, is caused by loss-of-function mutations in the *KCNJ2* gene (Jongsma and Wilders 2001; Perez-Riera et al. 2021). ATS is classified as Long QT Syndrome 7 (LQT7) (Tristani-Firouzi et al. 2002). Because it impedes myocellular repolarization, as individuals display an extended QT interval on their electrocardiograms (ECGs) (Tristani-Firouzi et al. 2002). But unlike other types of LQTS, it causes distinct ventricular arrhythmias with a lower susceptibility to sudden cardiac death, and it also interferes with other systems in addition to the heart's electrical system (Tristani-Firouzi et al. 2002). However, subsequent clinical findings conclude that the Q-U interval is markedly prolonged instead of the QT interval, so some researchers prefer to name this disease ATS type 1 (AST1) rather than LQT7 (Adler et al. 2020; Delannoy et al. 2013; Moreno-Manuel et al. 2023; Nguyen et al. 2013; Zhang et al. 2005b). An LQTS-specific genetic study from 2020 shows that the evidence to classify ATS as LQTS is limited (Adler et al. 2020). ATS1 is a rare autosomal dominant inherited disorder that accounts for 60% of the ATS incidence (Veerapandiyan et al. 2018). ATS2 which is caused by the mutation of *KCNJ5*, coding for a G protein-activated inward-rectifier channel, accounts for 15%, the remaining ATS cases are not yet linked to a genetic variant (Kokunai et al. 2014; Perez-Riera et al. 2021).

ATS shares some phenotypes with the catecholaminergic polymorphic ventricular tachycardia (CPVT) (Le Tanno et al. 2021; Nguyen and Ferns 2018). Mutations (G144D, T305S, R67Q, V227F, R82W) in the *KCNJ2* gene were identified in individuals who had the clinical phenotype features of CPVT (Kalscheur et al. 2014; Kimura et al. 2012; Tester et al. 2006). However, a gene curation expert panel (GCEP) deemed that *KCNJ2* gene mutations scored with limited evidence as single gene causes for CPVT and classified the *KCNJ2* gene as “disputed” (Walsh et al. 2022). The GCEP prefers that these *KCNJ2* mutations were more accurately corresponded to a cardiac-restricted expression of ATS (Walsh et al. 2022).

There are no standardized treatment methods or guidelines for ATS (Moreno-Manuel et al. 2023). Medication management such as beta-blockers or anti-arrhythmic drugs may be prescribed to help control abnormal heart rhythms and calcium channel blockers may be used to manage the potassium imbalances that can occur in ATS1 (Kostera-Pruszczyk et al. 2015; Perez-Riera et al. 2021; Sansone and Tawil 2007). For specific symptoms, like periodic paralysis caused by low serum potassium levels, daily potassium supplementation will be beneficial (Statland et al. 2018). The elevated serum potassium level also benefits patients who suffer a long QT interval at the same time (Sansone and Tawil 2007). Because of the rarity of ATS (1: 500,000–2,000,000), it is very difficult to collect sufficient patients for clinical trials (Barron-Diaz et al. 2020). Therefore, animal models or cell models of ATS are highly desirable. Even though various treatments have been reported in the medical literature, they seldom target K_IR_2.1 channels. Class 1c antiarrhythmic drugs like flecainide and propafenone together with beta-blockers are used to treat ATS1 (Barajas-Martinez et al. 2011; Delannoy et al. 2013). Interestingly, flecainide and propafenone have direct drug-channel interactions with the K_IR_2.1 channel by binding with Cysteine 311 (Cys311), thereby increasing I_K1_ current by reducing the binding affinity of polyamine (Caballero et al. 2010; Gomez et al. 2014). However, the efficacy of these two drugs was controversial in the clinical setting (Barajas-Martinez et al. 2011; Bienias et al. 2018; Junker et al. 2002; Nguyen and Ferns 2018). Researchers have been exploring various compounds and approaches that modulate the activity of K_IR_2.1 channels, aiming to find out some potential therapeutic methods. Zacopride was originally used as an antiemetic agent, but its effects on the cardiovascular system were getting noticed (Smith et al. 1989). Zacopride acts as a K_IR_2.1 channel opener leading to hyperpolarization, shortening the APD, and suppressing aconitine, acute ischemic and reperfusion-induced arrhythmias in rat (Liu et al. 2021, 2012; Zhai et al. 2017). Unfortunately, the Zacopride work is still hampered by a case of scientific misconduct (Korte and Van Der Heyden 2017). The small-molecule drug BGP-15 was reported to stabilize the I_K1_ current amplitude after cells suffered PIP_2_ depletion (Handklo-Jamal et al. 2020a). BGP-15 probably can regulate the level, availability, or localization of PIP_2_, thereby stabilizing the open state of the K_IR_2.1 channel (Handklo-Jamal et al. 2020a). Tetramisole was shown to increase the I_K1_ current by facilitating the forward trafficking of the K_IR_2.1 channel, deactivation PKA signaling, and restoring the Ca^2+^ balance (Liu et al. 2022). A desmosome protein Plakophilin 4 (PKP4) was reported as an IK_1_ up regulator by BioID and patch clamp analysis (Park et al. 2020). These potential K_IR_2.1 positive modulators of I_K1_ (AgoKirs (Van Der Schoor et al. 2020)) are interesting candidates and starting points in generating pharmacotherapy to relieve or resolve symptoms of ATS (Table 1).

**Table 1:** Drugs and compounds modifying K_IR_2.1 carried I_K1_

S.no	Drug/compound	Effects on I_K1_	Working mechanism	in clinical use	References
	*AgoKirs*				
1	Propafenone	I_KIR2.1_ EC_50_ 12 nM	Reduce the binding affinity of polyamine by direct bind with Cys311 on the K_IR_2.1 channel	Yes	(Gomez et al. 2014; Hii et al. 1991)
2	Flecainide	I_KIR2.1_ EC_50_ 0.4 μM	Reduce the binding affinity of polyamine by direct bind with Cys311 on the K_IR_2.1 channel	Yes	(Basza et al. 2023; Caballero et al. 2010)
3	Zacopride	I_K1_ 28—40 μM	Associate with CaMKII, PKA Signalling	No	(Elnakish et al. 2017; Liu et al. 2021; Zhang et al. 2013a)
4	BGP-15	I_K1_ 30–45% at 50 μM	Presumed PIP_2_ interference	No	(Handklo-Jamal et al. 2020b)
5	Tetramisole	I_K1_ EC_50_ approx. 10 μM	Facilitate K_IR_2.1 channel forward trafficking, deactivation PKA signalling, restoring the Ca^2+^ balance	Yes	(Liu et al. 2022; Thienpont et al. 1969)
	*AntaKirs*				
6	Carvedilol	I_KIR2.1_ IC_50_ > 50 μM	Presumed PIP_2_ interference	Yes	(Ferrer et al. 2011; Turco et al. 2023)
7	Quinidine	I_KIR2.1_ IC_50_ approx.290 μM	Prevent re-entry associated with the heterozygous D172N condition; Pore block and PIP_2_ interference	Yes	(Koepple et al. 2017; Li et al. 2021a; Luo et al. 2017a)
8	Quinacrine	I_KIR2.1_ IC_50_ 65 μM	Direct pore block and PIP_2_ interference	Yes	(Lopez-Izquierdo et al. 2011a)
9	Chloroquine	I_K1_ IC_50_ 0.69 μM	Direct pore block and PIP_2_ interference	Yes	(Martinez et al. 2020; Noujaim et al. 2010)
10	Gambogic acid	I_KIR2.1_ IC_50_ 4.8 μM	Change the K^+^ channel membrane microenvironment, pore block and PIP_2_ interference	No	(Scherer et al. 2017; Zaks-Makhina et al. 2009)
11	Mefloquine	I_KIR2.1_ IC_50_ > 30 μM	Presumed PIP_2_ interference	Yes	(Lopez-Izquierdo et al. 2011b; Ter Kuile et al. 1995)
12	ML-133	I_KIR2.1_ IC_50_ 1.8 μM	Reduce K^+^ conductance	No	(Sanson et al. 2019; Wang et al. 2011)
13	Pentamidine	I_KIR2.1_ IC_50_ 170 nM	Pore block, interacting with E224, D259, E299	Yes	(De Boer et al. 2010b; Smith et al. 1991)
14	PA-6	I_KIR2.1_IC_50_ 12–15 nM	Pore block, interacting with E224 and E299	No	(Takanari et al. 2013)
15	Tamoxifen	I_KIR2.1_ IC_50_ 0.93 μM	presumed PIP_2_ interference	Yes	(Ponce-Balbuena et al. 2009; Wibowo et al. 2016)
16	Thiopental	I_KIR2.1_ IC_50_ approx. 30 μM	Presumed PIP_2_ interference and potential R218 interaction	Yes	(Bellante et al. 2016; Lopez-Izquierdo et al. 2010)

### Short QT syndrome type 3

Short QT syndrome (SQTS) is a rare genetic disorder characterized by an abnormally short QT interval on the ECG, indicating a shortening of the depolarization-repolarization cycle for each heartbeat (Dewi and Dharmadjati 2020). The shortened QT interval can disrupt the heart's normal electrical activity, potentially leading to cardiac events, and increasing the risk of life-threatening arrhythmias, such as ventricular fibrillation and sudden cardiac arrest (Dewi and Dharmadjati 2020; Hancox et al. 2023; Kim et al. 2021). Schwartz's score helps in diagnosing SQTS and its subtypes (Dewi and Dharmadjati 2020). There are now 6 subtypes of SQTS recognized, including SQTS3, which is associated with a gain-of-function mutation in the *KCNJ2* gene (Dewi and Dharmadjati 2020). The first reported mutation site is at amino acid 172, mutated from aspartic acid to asparagine (D172N) (Priori et al. 2005). D172 is an important binding site for polyamines, therefore the D172N substitution in the *KCNJ2* gene (gain-of-function mutation) can lead to abnormal functioning of K_IR_2.1 channels, increasing the outward current and subsequently inducing a shortened QT interval (Anumonwo and Lopatin 2010; Du et al. 2021; Priori et al. 2005). The *KCNJ2* gain-of-function mutations M301K and E299V were found in an 8-year-old girl and an 11-year-old boy, respectively (Deo et al. 2013; Hattori et al. 2012). Both showed extremely short QT intervals together with paroxysmal atrial fibrillation (AF) (Deo et al. 2013; Hattori et al. 2012). Another SQTS3-related gain-of-function mutation K346T was reported in 2014 (Ambrosini et al. 2014). F58S was reported as a gain-of-function mutation of K_IR_2.1 in 2018, the increased I_K1_ decreased the neuro excitability and shorted the heart QT interval at a borderline level so that the patient was only diagnosed as an autism spectrum disorder (ASD) (Binda et al. 2018).

The first SQTS was reported in 2000 and SQT3 was first described in 2005. Since, specific treatment of SQTS is still poorly defined (Gussak et al. 2000; Priori et al. 2005; Rudic et al. 2014). The implantable cardioverter defibrillator (ICD) is used as the first-line therapy in SQTS but with an increased risk of inappropriate shock (Dewi and Dharmadjati 2020). The Class Ia antiarrhythmic drug quinidine is regarded as the most effective pharmacological therapy in SQTS patients (Dewi and Dharmadjati 2020; Hancox et al. 2023; Rudic et al. 2014). Quinidine can prolong the APD of the ventricular cells, increase the effective refractory period (ERP), and reduce the susceptibility of ventricular tissues associated with SQT3 (Luo et al. 2017a). In addition, chloroquine (CQ) prolongs the APD by reducing I_K1,_ and probably other repolarizing currents (I_Kr_, I_to_), which may be a potential agent for SQT3 treatment in the future (Luo et al. 2017b; Szendrey et al. 2021; Wagner et al. 2010). On a longer time-scale, CQ application results in lysosomal accumulation of K_IR_2.1 proteins (Jansen et al. 2008). More specific I_K1_ inhibiting drugs (AntaKirs) may find a place in SQT3 pharmacotherapy.

### Familial atrial fibrillation

AF is characterized by an irregular and often accelerated heart rhythm with a high risk of stroke, heart failure, and various other complications related to the heart and currently affects over 33 million individuals worldwide (Al-Khatib 2023; Wijesurendra and Casadei 2019). Several known risk factors are high blood pressure, sleep disorders, diabetes, obesity, chronic lung diseases, coronary artery disease, congenital defects, etc. (Benjamin et al. 2021; Buch et al. 2003; Christophersen and Ellinor 2016; Chugh et al. 2014; Lau et al. 2017). Between 5 and 15% of patients with AF have a familial predisposition, and there are many mutations related to Familial Atrial Fibrillation (FAF) (Christophersen and Ellinor 2016; Darbar et al. 2003). Xia et al. first analyzed the distribution of the *KCNJ2* gene in relatives of Chinese patients with FAF in 2005 (Xia et al. 2005). The mutation V93I was found in all 30 unrelated kindreds, whereas none was abnormal in 420 unrelated healthy Chinese individuals (Xia et al. 2005). Electrophysiologic studies also confirmed the increase of the outward currents generated by V93I-K_IR_2.1 channels The enhanced activity of K_IR_2.1 channels resulted in a shorter APD, leading to the development of AF. However, in a recent study, one V93I carrier shows an evident QT prolongation, which indicates that its clinical appearance is not so consistent (Zaklyazminskaya et al. 2022).

The treatment and prevention of AF was summarized by Al-Khatib (Al-Khatib 2023). Ongoing studies have explored gene therapy approaches aimed at restoring normal K_IR_2.1 channel functions. MicroRNA-26 (miR-26) was downregulated in *KCNJ2*-upregulated AF animals and patients, and the knockdown, inhibition, or binding-site mutation of miR-26 enhanced the expression of *KCNJ2*, establishing that *KCNJ2* is a miR-26 target (Luo et al. 2013). Long noncoding RNA TCONS-00106987 (lncRNA TCONS-00106987) was reported to increase the expression of the K_IR_2.1 channel proteins in rabbit models by endogenously competing with miR-26, suggesting that the mutual regulation between lncRNA and miRNA can be a potential therapeutic target for AF (Du et al. 2020). The pentamidine analogue PA-6 (Table 1) was presented as a specific I_K1_ inhibiting compound able to terminate AF in a goat model of recently induced AF, but not in patient dogs with long-lasting AF (Ji et al. 2017a, b; Szatmari et al. 2018).

### Acquired diseases associated with disturbed K_IR_2.1 channel functioning

Besides genetic diseases, altered K_IR_2.1 channel function or expression levels can also contribute to, or at least associate with, the development of some acquired diseases. Upregulation of I_K1_ was found to be involved in chronic atrial fibrillation (Dobrev et al. 2002; Zhang et al. 2005a). Many studies (clinical, animal, and computer simulation) have demonstrated that the weakening of I_K1_ which contributes to AP prolongation, is an important mechanism contributing to the development of arrhythmias in heart failure (Akar et al. 2005; Husti et al. 2021; Jian et al. 2022; Kaab et al. 1996; Li et al. 2004). Decreased I_K1_ was also associated with myocardial infarction, hypotrophy, and reperfusion arrhythmias (Aimond et al. 1999; Li et al. 2019; Liu et al. 2021; Roman-Campos et al. 2009). Correction of K_IR_2.1 channel function by pharmacologic or other molecular means will be beneficial in alleviating these diseases. However, the safety of the I_K1_ modulators should never be eliminated. First, since in the heart lengthening or shortening the APD duration may lead to life-threatening arrhythmias. Secondly, K_IR_2.1 channels have many functions in other organ systems and tissues which should not be compromised by efforts to normalize K_IR_2.1 function in the heart. This latter safety issue is likely a bigger problem in acquired than in genetically originated diseases.

## Future development of K_IR_2.1 pharmacology

With advancements in understanding the role of the K_IR_2.1 channel in various diseases, there may be an increased focus on developing targeted therapies that modulate the activity of the channel. This could involve the design of K_IR_ specific drugs and targeted gene therapy. The K_IR_ drugs should target K_IR_2.1 channels to enhance (AgoKir) or inhibit K_IR_2.1 (AntaKir) function without further interactions, depending on the therapeutic goal.

### Drugs targeting PIP_2_

PIP_2_ acts as a signaling molecule and as indicated above is involved in the modulation of the K_IR_2.1 channels, i.e. the presence of PIP_2_ in the cell membrane is necessary for stabilizing the open state of K_IR_2.1 channels (D'avanzo et al. 2010; Xie et al. 2008). When the affinity between PIP_2_ and K_IR_2.1 channels is reduced, K_IR_2.1 channels tend to lose function, leading to changes in membrane potential and cellular excitability (Donaldson et al. 2003; Xie et al. 2008). PIP_2_ binding affinity also determines the sensitivity of other K_IR_2.1 modulators like pH, PKC, and Mg^2+^ (Du et al. 2004; Gada and Logothetis 2022). Many studies have shown that the mutation defects affecting PIP_2_ binding constitute a major pathogenic mechanism of ATS (Choi et al. 2007; Cruz et al. 2023; De Los Monteros et al. 2022; Donaldson et al. 2003; Handklo-Jamal et al. 2020a, b; Lopes et al. 2002; Tan et al. 2013). Developing drugs that enhance PIP_2_-K_IR_2.1 channel interaction and thereby activate I_K1_ to alleviate ATS will be promising. In this direction, the small molecular BGP-15 (Table 1) showed increased PIP_2_ sensitivity in ATS variants (Handklo-Jamal et al. 2020a). A recent computer simulation study showed that the severity of the ATS mutation directly correlates with the electrostatic forces of the transmembrane PIP_2_ binding region (De Los Monteros et al. 2022). This may point to a way to relieve arrhythmias by neutralizing the positive charge in the K_IR_2.1 channel pore. By contrast, excessive K_IR_2.1 channel activity can also be corrected by blocking PIP_2_ mediated activation of the channel (Lopez-Izquierdo et al. 2011b; Ponce-Balbuena et al. 2009; Ruddiman et al. 2023). Drugs targeting PIP_2_, and more specifically the PIP_2_ dependent K_IR_2.1 activity, will be an interesting pharmacotherapeutic option for patients who suffer from diseases caused by abnormal K_IR_2.1 activities like ATS.

### Drugs interfering with channel trafficking

Disruptions and enhancement in channel trafficking can lead to alterations in K_IR_2.1 ion channel expression on the cell surface, which can lead to diseases like ATS, and SQT3 (Ambrosini et al. 2014; Bendahhou et al. 2003). In the context of drug development, targeting channel trafficking represents a potential strategy to modulate ion channel activity to treat certain channelopathies or other related disorders. There are various ways that drugs can interfere with channel trafficking, including promoting or inhibiting channel forward or backward trafficking, modulating intracellular accumulation, regulating endocytosis and recycling, and so on. The proteins mentioned in Fig. 3 (Golgin-97, AP-1, GRIF-1, SAP-97, ESCRT) are necessary for the forward trafficking, backward trafficking, or recycling of the K_IR_2.1 channel. Drugs that specifically interfere with these proteins' function with respect to their role in K_IR_2.1 trafficking, may indirectly help to correct the function of abnormal K_IR_2.1 channel function. For example, tetramisole showed a high selective affinity with the K_IR_2.1 channel and increased I_K1_, one of its mechanisms is promoting K_IR_2.1 forward trafficking through the upregulation of SAP-97 (Liu et al. 2022).

### Drugs enhancing K^+^ conductance

The potassium conductance of the K_IR_2.1 channel is primarily determined by its structure and the arrangement of specific amino acids within the channel pore (Fernandes et al. 2022b). Drugs that enhance the conductance of the K_IR_2.1 channel can increase its preference for K^+^, potentially allowing more K^+^ to pass through while blocking or restricting the movement of other ions. Potassium channel openers are a class of drugs that have the potential to increase the conductance of K^+^. This increase in I_K1_ may benefit patients who suffer from ATS or HF in the future.

### Some other specific channel agonists or inhibitors

There are several K_IR_2.1 modulators, or at least compounds that include K_IR_2.1 modulatory behavior amongst other properties. For example, propafenone, flecainide, aldosterone, sildenafil, etc. (Alexandre et al. 2015; Caballero et al. 2010; Gomez et al. 2014; Iijima et al. 2023). These compounds probably will generate a lot of side effects due to their multifunctional characteristics if they work as K_IR_2.1 modulators. The promising modulators will specifically target the channel without interfering with other channels. Studies showed that zacopride can promote I_K1_ without interfering with voltage-gated Na^+^ current, L-type Ca^2+^ current, transient outward K^+^ current, sustained outward K^+^ current, delayed rectifier K^+^ current, and current generated through Na^+^–Ca^2+^ exchanger and Na^+^–K^+^ pump (Lin et al. 2020; Liu et al. 2012). Gambogic acid is a specific, slow K_IR_2.1 inhibitor by changing the K^+^ channel membrane microenvironment (Zaks-Makhina et al. 2009). ML-133 was reported as a potent specific K_IR_2.x inhibitor, reducing K^+^ conductance through the channel by interfering with D172 and I176 on the M2 segment of the channel (Wang et al. 2011). A study in 2019 showed that the Hill coefficient of ML-133 is 2.6 (> 1) meaning that ML133 probably has a cooperative mechanism of action which is still unknown (Sanson et al. 2019). Drugs increasing or decreasing the binding affinity of polyamines or Mg^2+^ can also be utilized for future therapeutic purposes. The discovery of specific K_IR_2.1 modulators provides a path for functional studies of I_K1_. More “next generation” K_IR_ channel modulators which are both potent and specific are needed, to achieve this, employing high-throughput screening of small-molecule libraries and utilizing medicinal chemistry (structure-based drug design) will help (Weaver and Denton 2021).

### Nutrigenomics

People have used food and plants as medicines since ancient times, as they know that food and the environment can interfere with an individual’s health conditions (Sales et al. 2014). The emergence of the field of nutrigenomics, which combines genomic science with nutrition, is a direct consequence of elucidating the interactions between genes and nutrients (Sales et al. 2014). Understanding this interaction process could lead to the prescription of specific diets for each individual. Nutrigenomics is a relatively new field in regulating K_IR_ channels, specific diets or variations in nutrient conditions have the potential to influence the expression or function of K_IR_ channels in tissues (Ferreira et al. 2023). Diets rich in cholesterol have been associated with increased membrane cholesterol content, which resulted in a decreased function of K_IR_2.1 channels (Ferreira et al. 2023; Yuan and Hansen 2023). Diets rich in phosphoinositide or inositol like liver, grains, and legumes could increase the expression level and functions of K_IR_2.1 channels by altering the levels of PIP_2_ in cells through the metabolite process (Ferreira et al. 2023). Diets rich in salt interfere with potassium handling, and even though no nutrition-related factors are reported in patients of ATS, special diets that are low in carbohydrates and Na^+^ were recommended for some cases (Ferreira et al. 2023; Welland et al. 2021). Diets rich in polyamines like cheese, meat, vegetables, etc. show prominent cardioprotective and neuroprotective effects (Madeo et al. 2018; Munoz-Esparza et al. 2019). Diets rich in Mg^2+^ like vegetables, nuts, seeds, etc. are necessary in some cases, as chronic Mg^2+^ deficiency could downregulate the expression of the K_IR_ 2.1 channel and cause a reduction of I_K1_ (Al Alawi et al. 2018; Shimaoka et al. 2020). The coupling between nutrient and K_IR_ channels is complex and can involve multiple mechanisms, more investigations are needed in this field.

### Gene therapy

*KCNJ2* mutations could cause AST, SQT3, FAF, and maybe some other diseases not currently detected. Gene analysis is already used in part of the cardiovascular diseases workup, it helps to make more accurate diagnoses (Di Toro et al. 2019; Vivekanandam et al. 2022; Xia et al. 2005). Developing gene therapy techniques allows for the direct manipulation of K_IR_2.1 channel expression or activity in specific tissues. Gene therapy helps to replace the mutant DNA with the wild-type DNA, leading to a normal function of the K_IR_2.1 channel.

## Conclusions

The functional K_IR_2.1 channel helps to stabilize the resting membrane potential and regulate the excitability of the heart. This channel plays important roles in the maintenance of normal heart rhythm and cell communication. All patients who suffered similar symptoms related to the *KCNJ2* gene should be offered a genetic diagnosis, that might further assist in individualized treatment. The development of drugs or gene therapy targeting the K_IR_2.1 channel is a complex and challenging process. Further research and clinical investigations are necessary to fully understand the complete range of diseases or conditions associated with the K_IR_2.1 channel.

## Data Availability

No datasets were generated or analysed during the current study.

## Abbreviations

AP/APD: Action potential/Action potential duration
AP-1: Adaptor protein 1
ATS: Anderson-Tawil syndrome
ASD: Autism spectrum disorder
CTD: Cytoplasmic domain
CPVT: Catecholaminergic Polymorphic Ventricular Tachycardia
CQ: Chloroquine
ERP: Effective refractory period
ER: Endoplasmic reticulum
ESCRT: Endosomal sorting complex required for transport
FAF/AF: Familial Atrial Fibrillation /Atrial Fibrillation
GRIF-1: γ-Aminobutyric acid type A receptor interacting factor-1
GFP: Green fluorescent protein
GCEP: Gene curation expert panel
HF: Heart Failure
I_K1_: Inward rectifier potassium current
K_IR_2.1, K_IR_2.2, K_IR_2.3: Inward rectifier potassium channel
KWGF: Human embryonic kidney (HEK)-293 cells that stably express C-terminal GFP-tagged murine K_IR_2.1
MVB: Multivesicular bodies
PKA: Protein kinase A
PKC: Protein kinase C
PKP4: Plakophilin 4
PIP_2_: Phosphatidylinositol-4,5-bisphosphate
RMP: Resting membrane potential
Rac1: Ras-related C3 botulinum toxin substrate 1
SAP-97: Scaffolding protein
SQT/LQT: Short QT syndrome / Long QT Syndrome
TMD: Transmembrane domain
